# From criminal interrogations to investigative interviews: a bibliometric study

**DOI:** 10.3389/fpsyg.2023.1175856

**Published:** 2023-06-19

**Authors:** Vincent Denault, Victoria Talwar

**Affiliations:** Department of Educational and Counselling Psychology, McGill University, Montreal, QC, Canada

**Keywords:** criminal interrogations, investigative interviews, bibliometrics, web of science, solving crimes, identifying perpetrators

## Abstract

This paper presents the results of a bibliometric study providing a comprehensive overview of the social science research conducted on criminal interrogations and investigative interviews since the 1900s. The objectives are to help researchers to further understand the research field, to better communicate research findings to practitioners, to help practitioners understand the breadth of scientific knowledge on criminal interrogations and investigative interviews, and to foster dialog between researchers and practitioners. To begin, after a brief description of Web of Science, we describe how we developed our database on criminal interrogations and investigative interviews. Then, we report the yearly evolution of articles, the journals where they were published, the research areas covered by this research field, as well as the authors, the institutions and the countries that published the most on a variety of topics related to criminal interrogations and investigative interviews. Finally, we present the most used keywords and the most cited articles, and examine the research on questionable tactics and techniques in the research field of criminal interrogations and investigative interviews. This paper ends with a critical look at the results, for the benefit of researchers and practitioners interested in criminal interrogations and investigative interviews.

## Introduction

Tactics and techniques for solving crimes and identifying perpetrators evolved throughout history. For a long time, they were based on anecdotal evidence and spiritual belief. For example, in ancient India, crime suspects, crime suspects could be asked to take a handful of rice and put it in their mouth. The crime suspects would then spit out the rice, and if it was dry, or mixed with blood, they were considered guilty of the crime (Trovillo, 1939). Around the same time, in ancient sacred scriptures of Hinduism, instructions were given on how to discover someone who wanted to poison others. “He does not answer questions, or they are evasive answers; he speaks nonsense, rubs the great toe along the ground, and shivers; his face is discolored; he rubs the roots of the hair with his fingers; and he tries by every means to leave the house” (Wise, 1845, p. 394).

The above are only two of many examples. But historical evidence is abundant. The observation of behaviors and physical features has long been central to tactics and techniques for solving crimes and identifying perpetrators. This continued during the Middle Ages. For example, trials by ordeal were based on the belief that God would hold harmless innocent individuals. A crime suspect was subjected to a physical test and depending on the healing of the injury then sustained, a decision was made. In one of them, the red-hot iron ordeal, following a religious ritual, the crime suspect had to walk a few steps with a piece of burning iron in his hand. The piece of burning iron was then removed, the hand was covered with a bandage, and a few days later, the bandage was removed, and the injury was examined. Depending on the healing, the decision of innocence or guilt was made (White, 1961; Pilarczyk, 1996). Bizarre practices have continued long after the Middle Ages, including during the 1,692 witchcraft trial in Salem, Massachusetts (Barry, 1994; Moriarty, 2001). Phrenology is another example of the weight given to observation in order to solve crimes and identify perpetrators. Popularized in the 19th-century, this technique claimed that scalp morphology was related to character traits (Stea et al., 2022), and was used to assess whether certain people might have a propensity to commit crimes (Berveling, 2021).

Besides the observation of behaviors and physical features, other tactics and techniques for solving crimes and identifying perpetrators assumed the human body reveals the truth. For example, during the 1900s, the ancestors of today’s polygraphs were developed. Based on the premise that specific physiological reactions are indicative of deception, the polygraph would later become central in several law enforcement organizations around the world, but still be subject to severe criticism (Grubin and Madsen, 2005). Polygraph “as used in many places, is nothing more than a psychological third degree aimed at extorting confessions as the old physical beatings were” (Lykken, 1998, pp. 28–29; see also Leo, 2008; Denault, 2014).

Throughout history, besides the assumption that the human body reveals the truth, tactics and techniques for solving crimes and identifying perpetrators advocated for extraction rather than observation of behaviors and physical features. Torture is a prime example. According to the United Nations (1984), torture refers to:

…any act by which severe pain or suffering, whether physical or mental, is intentionally inflicted on a person for such purposes as obtaining from him or a third person information or a confession, punishing him for an act he or a third person has committed or is suspected of having committed, or intimidating or coercing him or a third person, or for any reason based on discrimination of any kind, when such pain or suffering is inflicted by or at the instigation of or with the consent or acquiescence of a public official or other person acting in an official capacity. It does not include pain or suffering arising only from, inherent in or incidental to lawful sanctions.

Torture has been used for thousands of years, and is still used today in dozens of countries, including the United States (Amnesty International, n.d.). The same holds for the use of physical coercion within in law enforcement. For example, in the United States, during the 1900s, physical coercion, also known as “third degree” tactics, were common among police forces (Kassin et al., 2010). This is until the courts began to narrow their scope. In 1936, for example, the United States Supreme Court (Brown v. Mississippi, 1936) forced law enforcement to stop using physical coercion, because from now on, confessions obtained under torture would not be admitted as evidence (Leo, 1992; Darmer, 2009), and in 1966, the United States Supreme Court required law enforcement to warn suspects in custody of their rights before interrogating them (Miranda v. Arizona, 1966).

The decline of physical coercion among law enforcement, however, was replaced by other questionable methods to extract confessions. Accusatorial methods using psychological manipulation and persuasion, notably the Reid method developed by John E. Reid and Fred Inbau in the 1940s (Kassin et al., 2010), grew in popularity. The assumption that the human body reveals the truth was part of the first step of the Reid method. It was claimed that behaviors helped determine if the suspect’s guilt “in the opinion of the investigator, seems definite or reasonably certain” (Inbau et al., 2013, p. 185), and if attempts to extract confessions should subsequently be made through the second step of the Reid method (Denault et al., 2020). However, accusatorial methods came under increasing criticism when researchers started to address them, and demonstrated, among other things, the danger of false confessions (Gudjonsson, 2021). A series of wrongful convictions has even led the UK to fundamentally change police practices. The PEACE (Preparation and Planning, Engage and Explain, Account, Closure, Evaluation) model, for example, was developed by psychologists, lawyers, and police officers, and aimed at eliciting information from victims, witnesses and suspects, rather than extracting confessions. More than 140,000 officers in England and Wales were trained in this model in the 1990s (Bearchell, 2010; Poyser and Milne, 2021). Law enforcement in the United States gradually followed, moving away from accusatorial methods and adopting rapport-building interviewing techniques (Mindthoff and Meissner, 2023). This change, however, was not widespread. Questionable methods to extract confessions are still integral to law enforcement around the world. This probably explains why the Principles on Effective Interviewing for Investigations and Information Gathering (also known as the Méndez Principles), an international initiative supported by the UN Human Rights Council, were translated in 10 languages. Those principles provide law enforcement “guidance on obtaining accurate and reliable information in full respect of the human rights and dignity of all, including through the implementation of legal and procedural safeguards in the first hours of police custody” (Association for the Prevention of Torture, n.d.).

However, despite the worldwide popularity of questionable methods to extract confessions, scientific data on criminal interrogations and investigative interviews is abundant. There are several handbooks on the subject (e.g., Williamson et al., 2013; Walsh et al., 2015; Lamb et al., 2018; Bull and Blandon-Gitlin, 2019; Barela et al., 2020). There are also several meta-analyses and reviews (e.g., Abbe and Brandon, 2013; van Ham et al., 2020; Akca et al., 2021; Lavoie et al., 2021; Meissner et al., 2023). Just recently, in 2021, Christian Meissner edited “What Works? Systematic Reviews and Meta-Analyses of the Investigative Interviewing Research Literature,” a special issue of Applied Cognitive Psychology.

Moreover, scientific data on criminal interrogations and investigative interviews goes far beyond theoretical issues. The implementation of evidence-based practices, for example, has been subject to extensive research (e.g., Milne et al., 2019; Risan et al., 2020; Brimbal et al., 2021; Brubacher et al., 2021; Nicol et al., 2023). However, despite the numerous publications, little is known about the structure of the research field of criminal interrogations and investigative interviews. And while historical evidence allows for a better understanding of how tactics and techniques to solve crimes and identify perpetrators evolved throughout history, to our knowledge, there is no data about collaboration patterns, thematic groups, research constituents, emerging trends, and other characteristics of a research field that advocates the importance of scientific data in developing better practices and in challenging unfounded and discredited tactics and techniques that a number of law enforcement organizations have turned to (e.g., Denault et al., 2020; Smith, 2020). This is not to be overlooked. All the more since the research field of criminal interrogations and investigative interviews fundamentally changed police practices.

Therefore, rather than waiting for historical evidence, this paper presents the results of a bibliometric study providing a comprehensive overview of the social science research conducted on criminal interrogations and investigative interviews since the 1900s. The objectives are to help researchers to further understand the research field, to better communicate research findings to practitioners, to help practitioners understand the breadth of scientific knowledge on criminal interrogations and investigative interviews, and to foster dialog between researchers and practitioners.

To achieve these objectives, the bibliometric study was conducted with the “Social Sciences Citation Index (SSCI)—1900-present” collection of Web of Science. This type of study offers the possibility to better understand the structure of a research field, including collaboration patterns, thematic groups, research constituents, and emerging trends (Donthu et al., 2021). Sugimoto and Larivière (2018) explain the relevance of a bibliometric study:

Bibliometrics are particularly useful when the amount of data exceeds human capabilities to process. For example, a reviewer is well equipped to make a judgment on a single document or small set of documents. An author can fairly easily ascertain the number of publications he or she produced. However, measurements of the production of an institution or country are harder to gauge. Furthermore, relational data—like citations are nearly impossible to manually analyze even at the level of an individual. Therefore, measurements of research have their greatest utility at scale— to bring into the light that which is not easily observed by the individual (p. 3-4).

To begin, after a brief description of Web of Science, the bibliographic database used to carry out our bibliometric study, we describe how we developed our database on criminal interrogations and investigative interviews. Then, similarly to Denault et al. (2022), we report the yearly evolution of articles, the journals where they were published, the research areas covered by this research field, as well as the authors, the institutions and the countries that published the most on a variety of topics related to criminal interrogations and investigative interviews. Finally, we present the most used keywords and the most cited articles, and examine the research on questionable tactics and techniques in the research field of criminal interrogations and investigative interviews. This paper ends with a critical look at the results, for the benefit of researchers and practitioners interested in criminal interrogations and investigative interviews.

## Methods

Created in the 1960s and maintained by Clarivate Analytics (Birkle et al., 2020), the Web of Science database features a massive amount of raw data about research publications and citations, and allows for large-scale bibliometric analysis. This raw data includes, for example, the document title, the author’s name and address, the publication’s name and year, language, and the number of times it has been cited. This database was chosen to create the corpus of articles for our bibliometric study on criminal interrogations and investigative interviews because it is “one of the most reliable publisher-independent global citation databases in the world” (Shamsi et al., 2022, p. 5992). The high quality of the metadata on Web of Science compared to Google Scholar was also considered (Mongeon and Paul-Hus, 2016; Sugimoto and Larivière, 2018). Not to mention similar studies on other research fields were conducted with Web of Science (e.g., Nadeau et al., 2018; Plusquellec and Denault, 2018; Dodier, 2019; Denault et al., 2022).

To create the corpus of articles for our bibliometric study, similarly to Denault et al. (2022), we first had to determine which concepts we wanted to include or to exclude. That is, the boundaries of the research field we wanted to investigate had to be set up. For example, although several papers about deception and lying are presented as useful to solve crimes and identify perpetrators, we decided not to focus on this issue, and not to use keywords related to deception and lying. Not to mention a bibliometric study on deception and lying has already been recently published (Denault et al., 2022). However, because of their similarity with investigative interviews, we decided to include forensic interviews in our search. Both involves gathering accurate and reliable information, but the former is with adults, and the latter is with children. However, we excluded motivational interviews, which aim at changing behavior posing health risks (Hall et al., 2012), and diagnostic interviews used by mental health professionals for establishing diagnostics (Andrews and Peters, 1998).

Then, after setting up the boundaries of the research field we wanted to investigate, we had to determine which articles to include or exclude. In other words, at what point is an article dealing with criminal interrogations and investigative interviews? This task is quite a challenge. For example, if an article addresses several subjects, including criminal interrogations and investigative interviews, should it be included or excluded? At what point do criminal interrogations and investigative interviews become the focus of an article? What criteria should be used to answer this question? The number of words, the research questions, the references cited? If the focus of an article is not criminal interrogations and investigative interviews, but is relevant and widely cited within this research field, should the article be included? If the focus of an article is criminal interrogations and investigative interviews, but is irrelevant and poorly cited within this research field, should the article be excluded? At what point an article is relevant and widely cited? At what point an article is irrelevant and poorly cited? Answering these questions involves making arbitrary decisions.

However, because consistency was a concern when creating the corpus of articles for our bibliometric study, we began a trial-and-error process to find the best search query, that is, a search query to automatically limit false positives (included articles that should have been excluded) and false negatives (excluded articles that should have been included), and to avoid having to manually identify articles to include or exclude. For example, unlike the term investigative interview which refers to a very specific concept, and results in few false positives and false negatives, the terms interrogation and interview yields a gargantuan number of false positives. For example, the term interview is widely used in abstracts referring to the qualitative methodology, and the term interrogation is used in a variety of ways unrelated to criminal interrogations and investigative interviews (e.g., “interrogation of diseases,” “interrogation of systems,” “interrogation of nanostructures”).

Therefore, following the trial-and-error process, on 14 February 2023, we conducted a three-step search query within the “Social Sciences Citation Index (SSCI)-1900-present” collection of Web of Science (see Table 1). First, we searched for articles with “investigative interview*” or “forensic interview*” in their titles, abstracts or keywords, both the authors’ keywords and Web of Science’s keywords (Keywords Plus). Second, we searched for articles with “interview*” or “interrogat*” or “confess*” in their titles or keywords, with one of the following words (police OR “law enforcement*” OR miranda OR “false confess*” OR suggestib* OR eyewitness OR eyewitnesses OR witness OR witnesses OR suspect OR suspects OR victim OR victims) in their titles, abstracts or keywords. Finally, we excluded articles with “motivation*” or “diagnostic*” in their titles, abstracts or keywords. This three-step search query yielded a total of 3,729 articles, but only articles and review articles were selected. Other formats such as book reviews and conference proceedings were excluded leading to a total of 3,423 publications. Finally, articles not written in English were excluded. This resulted in a total of 3,347 publications. Following previous bibliometric studies (e.g., Nadeau et al., 2018; Plusquellec and Denault, 2018; Dodier, 2019; Denault et al., 2022), this three-step search query was deemed to be good trade-off for getting the widest amount of results while limiting false positives and false negatives, although the emphasis on criminal interrogations and investigative interviews may vary from article to article.

**Table 1:** Parameters of the research in Web of Science.

CORE COLLECTION: Social Sciences Citation Index (SSCI)— 1900-present
(TS = (“investigative interview*” OR “forensic interview*”) OR ((TI = (interview* OR interrogat* OR confess*) OR AK = (interview* OR interrogat* OR confess*) OR KP = (interview* OR interrogat* OR confess*)) AND TS = (police OR “law enforcement*” OR miranda OR “false confess*” OR suggestib* OR eyewitness OR eyewitnesses OR witness OR witnesses OR suspect OR suspects OR victim OR victims))) NOT TS = (motivation* OR diagnostic*)
Results: 3729
[and] DOCUMENT TYPE: Articles AND Review Articles
Results: 3423
[and] LANGUAGE: English
Results: 3347

The Full Record and Cited References of those 3,347 publications were downloaded in a TAB Delimited File, 500 at a time (the limit allowed by Web of Science), and were combined in a single file for analysis in Microsoft Excel and VOS Viewer. Subsequently, a total of 14 articles were removed because their author was unknown, and 74 articles because their publication year was also unknown. Finally, the corpus of articles for our bibliometric study on criminal interrogations and investigative interviews featured a total of 3,259 articles in English.

## Results

The results of our bibliometric study are divided in nine sections. To begin, we report the yearly evolution of articles on criminal interrogations and investigative interviews, the Top 15 journals and the Top 15 research areas, as well as the Top 15 authors, the Top 15 institutions and the Top 15 countries that published the most on a variety of topics related to criminal interrogations and investigative interviews. Finally, we present the Top 15 most used keywords and the Top 15 most cited articles, and examine the research on questionable tactics and techniques, namely Kinesic Interview, Synergology, Scientific Content Analysis (SCAN), Behavior Analysis Interview (BAI), and Reid Technique within the research field of criminal interrogations and investigative interviews.

### The decades

As shown in Table 2, the highest number of articles about criminal interrogations and investigative interviews were published during the last decade (2010–2019) with a total of 1,507 articles, that is, 46.24% of all articles published since 1900, the first year covered by the “Social Sciences Citation Index (SSCI)—1900-present” collection of Web of Science. Half as many articles were published in the previous decade (2000–2009). Considering the number of articles per decades, the research field of criminal interrogations and investigative interviews took off strongly in the 1990s, with more than 96% of the articles published since then, and has grown substantially up to this day.

**Table 2:** Articles per decades.

Decades	Number of articles	Percentage of all articles
1900–1909	0	0.00
1910–1919	1	0.03
1920–1929	0	0.00
1930–1939	2	0.06
1940–1949	1	0.03
1950–1959	4	0.12
1960–1969	36	1.10
1970–1979	25	0.77
1980–1989	52	1.60
1990–1999	321	9.85
2000–2009	720	22.09
2010–2019	1,507	46.24
2020-present	590	18.10

### The journals

The 3,259 articles in our database on criminal interrogations and investigative interviews were published in more than 700 different journals. The Top 15 journals where they were published are presented in Table 3. The first place goes to Applied Cognitive Psychology with an Impact Factor of 2.360, and 7.09% (*n =* 231) of the articles in our database, closely followed by Psychology Crime & Law, at the second place with an Impact Factor of 1.752, and 6.20% (*n =* 202) of the articles in our database. Law and Human Behavior and Child Abuse & Neglect take the third and fourth place, respectively with an Impact factor of 3.870 and 4.863, and 4.45% (*n =* 145) and 3.84% (*n =* 125) of the articles in our database.

**Table 3:** Top 15 journals.

	Journals	Number of articles	IF (2021)	Percentage of all articles
1	Applied Cognitive Psychology	231	2.360	7.09
2	Psychology Crime & Law	202	1.752	6.20
3	Law and Human Behavior	145	3.870	4.45
4	Child Abuse & Neglect	125	4.863	3.84
5	Legal and Criminological Psychology	92	1.756	2.82
6	Psychiatry Psychology and Law	84	1.247	2.58
7a	Personality and Individual Differences	80	3.950	2.45
7b	Psychology Public Policy and Law	80	3.317	2.45
8	Journal of Investigative Psychology and Offender Profiling	71	1.119	2.18
9	Behavioral Sciences & The Law	63	2.568	1.93
10	Frontiers in Psychology	49	4.232	1.50
11	Journal of Child Sexual Abuse	45	1.872	1.38
12	Journal of Interpersonal Violence	39	2.621	1.20
13	Journal of Criminal Law & Criminology	38	2.184	1.17
14	Memory	32	2.519	0.98
15	Journal of Experimental Child Psychology	29	2.547	0.89

Interestingly, 7 journals in the Top 15 journals addressing issues of criminal interrogations and investigative interviews (Applied Cognitive Psychology; Frontiers in Psychology; Journal of Experimental Child Psychology; Law and Human Behavior; Legal and Criminological Psychology; Personality and Individual Differences; Psychology Crime & Law) are in the Top 15 journals addressing issues of deception and lying (Denault et al., 2022). This suggest that both research fields appeal to similar readerships. In addition, 3 journals in the Top 15 journals addressing issues of criminal interrogations and investigative interviews (Child Abuse & Neglect, Journal of Child Sexual Abuse, Journal of Experimental Child Psychology) focus on children. This suggests that the scientific community is concerned about children.

### The research areas

As shown in Table 4, almost two third of articles (*n =* 2038) about criminal interrogations and investigative interviews were published in journals covering Psychology related topics. Journals covering Government & Law (*n =* 1,031) and Criminology & Penology (*n =* 827) related topics have also made a significant contribution to the literature on criminal interrogations and investigative interviews. Furthermore, scientific knowledge about criminal interrogations and investigative interviews is enriched by studies done in several other research areas, including social work, linguistics, communication, and sociology, undoubtedly giving this research field an interdisciplinary dimension. It should be noted that journals indexed in Web of Science can be assigned to multiple research areas (Denault et al., 2022).

**Table 4:** Top 15 research areas.

	Research areas	Number of articles	Percentage of all articles
1	Psychology	2038	62.53
2	Government & Law	1,031	31.64
3	Criminology & Penology	827	25.38
4a	Psychiatry	297	9.11
4b	Family Studies	297	9.11
5	Social Work	210	6.44
6	Linguistics	107	3.28
7	Health Care Sciences & Services	103	3.16
8	Public, Environmental & Occupational Health	73	2.24
9	Communication	65	1.99
10	Business & Economics	57	1.75
11	Sociology	54	1.66
12	Rehabilitation	44	1.35
13	Education & Educational Research	42	1.29
14	Nursing	37	1.14
15	Biomedical Social Sciences	33	1.01

### The authors

More than 4,500 different authors contributed the 3,259 articles of our database on criminal interrogations and investigative interviews. As shown in Table 5, the author who published the most in this research field, regardless of authorship rank (e.g., first, second, third author, etc.), is Martine B. Powell (*n* = 115) from Griffith University, the founding director of the Centre for Investigative Interviewing, with 3.53% of all articles. Gisli H. Gudjonsson (*n* = 111) from King’s College London and Michael E. Lamb (*n* = 104) from the University of Cambridge follow closely behind at the second and third place, respectively with 3.41 and 3.19% of all articles. It should be noted that the number of articles is not the only way to measure the impact of authors within a research field. For example, Saul M. Kassin, from CUNY John Jay College of Criminal Justice, is at the 8th position, but his average number of citations per article is higher than authors with more articles than him, and he authored four of the Top 15 most cited articles on criminal interrogations and investigative interviews (see Table 6). In other words, the impact of authors within a research field can be measured in a variety of ways, including the number of articles, the average the number of citations per article, and the number of most-cited articles. Although it should be noted that in some cases authors may also be cited frequently because of repeated criticisms and scholarly debates.

**Table 5:** Top 15 authors.

	Authors	Number of articles	Percentage of all articles	Total number of citations	Average number of citations per article
1	Martine B. Powell	115	3.53	1810	15.74
2	Gisli Gudjonsson	111	3.41	3,664	33.01
3	Michael E. Lamb	104	3.19	4,854	46.67
4	Aldert Vrij	86	2.64	2,946	34.26
5	Par Anders Granhag	64	1.96	2,106	32.91
6	Ray Bull	54	1.66	2,443	45.24
7a	Ronald P. Fisher	51	1.56	3,217	63.08
7b	Rebecca Milne	51	1.56	1854	36.35
8	Saul M. Kassin	46	1.41	3,569	77.59
9a	Irit Hershkowitz	45	1.38	2,589	57.53
9b	Lorraine Hope	45	1.38	671	14.91
9c	Thomas D. Lyon	45	1.38	650	14.44
10	Carmit Katz	43	1.32	595	13.84
11a	Jon Fridrik Sigurdsson	42	1.29	1,003	23.88
11b	Sonja P. Brubacher	42	1.29	426	10.14
12	Samantha Mann	35	1.07	1,178	33.66
13	Sharon Leal	31	0.95	889	28.68
14	Fiona Gabbert	30	0.92	586	19.53
15a	Leif A. Strömwall	29	0.89	1,184	40.83
15b	Yael Orbach	29	0.89	2,385	82.24

**Table 6:** Top 15 most cited articles.

	Articles	Citation counts
1	Harrison, Y., and Horne, J. A. (2000). The impact of sleep deprivation on decision making: A review. *Journal of Experimental Psychology-Applied, 6*(3), 236–249.	741
2	Kassin, S. M., Drizin, S. A., Grisso, T., Gudjonsson, G. H., Leo, R., A., and Redlich, A. D. (2010) Police-Induced Confessions: Risk Factors and Recommendations. *Law and Human Behavior, 34*(1), 3–38.	385
3	London, K., Bruck, M., Ceci, S. J., and Shuman, D. W. (2005) Disclosure of child sexual abuse: What does the research tell us about the ways that children tell? *Psychology Public Policy and Law, 11*(1), 194–226.	336
4	Lamb, Michael, E., Orbach, Y., Hershkowitz, I., Esplin, P. W., and Horowitz, D. (2007). A structured forensic interview protocol improves the quality and informativeness of investigative interviews with children: A review of research using the NICHD Investigative Interview Protocol. *Child Abuse & Neglect, 31*, 1,201–1,231.	332
5	Fink, L. A., Bernstein, D., Handelsman, L., Foote, J., and Lovejoy, M. (1995). Initial reliability and validity of the childhood trauma interview - A new multidimensional measure of childhood interpersonal trauma. *American Journal of Psychiatry, 152*(9), 1,329–1,335.	323
6	Bruck, M., and Ceci, S. J. (1999). The suggestibility of children’s memory. *Annual Review of Psychology, 50*, 419–439.	321
7	Kassin, S. M., and Kiechel, K. L. (1996). The social psychology of false confessions: Compliance, internalization, and confabulation. *Psychological Science, 7*(3), 125–128.	295
8a	Orbach, Y., Hershkowitz, I., Lamb, M. E., Sternberg, K. J., Esplin, P. W., & Horowitz, D. (2000). Assessing the value of structured protocols for forensic interviews of alleged child abuse victims. *Child Abuse & Neglect, 24*(6), 733–752.	285
8b	Memon, A., Meissner, C. A., and Fraser, J. (2010). The cognitive interview: A meta-analytic review and study space analysis of the past 25 years. *Psychology Public Policy and Law, 16*(4), 340–372.	285
9a	Exline, J. J., Worthington, E. L., Hill, P., McCullough, M. E. (2003). Forgiveness and justice: A research agenda for social and personality psychology*. Personality and Social Psychology Review, 7*(4), 337–348.	270
9b	Gudjonsson, G. H. (1984). A new scale of interrogative suggestibility. *Personality and Individual Differences, 5*(3), 303–314.	270
10	Meissner, C. A., and Kassin, S. M. (2002). He′s guilty!: Investigator bias in judgments of truth and deception. *Law and Human Behavior, 26*(5), 469–480.	264
11	Kassin, S. M. (2005) On the psychology of confessions - Does innocence put innocents at risk? *American Psychologist, 60*(3), 215–228.	252
12	Chard, K. M. (2005). An evaluation of cognitive processing therapy for the treatment of posttraumatic stress disorder related to childhood sexual abuse. *Journal of Consulting and Clinical Psychology, 73*(5), 965–971	249
13	Vrij, A., Mann, S. A., Fisher, R. P., Leal, S., Milne, R., and Bull, R. (2008). Increasing cognitive load to facilitate lie detection: The benefit of recalling an event in reverse order. *Law and Human Behavior, 32*(3), 253–265.	234
14	Vrij, A. (2005). Criteria-Based Content Analysis: A Qualitative Review of the First 37 Studies. *Psychology, Public Policy, and Law, 11*(1), 3–41.	222
15	Geiselman, R. E., Fisher, R. P., MacKinnon, D. P., and Holland, H. L. (1985). Eyewitness memory enhancement in the police interview: Cognitive retrieval mnemonics versus hypnosis. *Journal of Applied Psychology, 70*(2), 401–412.	217

Furthermore, it should be remembered that we used the “Social Sciences Citation Index (SSCI)—1900-present” collection of Web of Science. Therefore, our bibliometric study focuses on a particular strand of research, that is, social sciences research on criminal interrogations and investigative interviews. However, legal scholars, for example, have extensively addressed issues of criminal interrogations and investigative interviews. Steven A. Drizin, from the Northwestern Pritzker School of Law, and Richard A. Leo, from the University San Francisco School of Law, are two of them. However, a number of law journals do not appear in the “Social Sciences Citation Index (SSCI)—1900-present.” The same holds for other disciplines. This is why our bibliometric study provides a rigorous, and novel, but inevitably incomplete picture of this research field. In other words, the research field of criminal interrogations and investigative interviews as a whole is likely much larger than the one described here.

**Figure 1:**
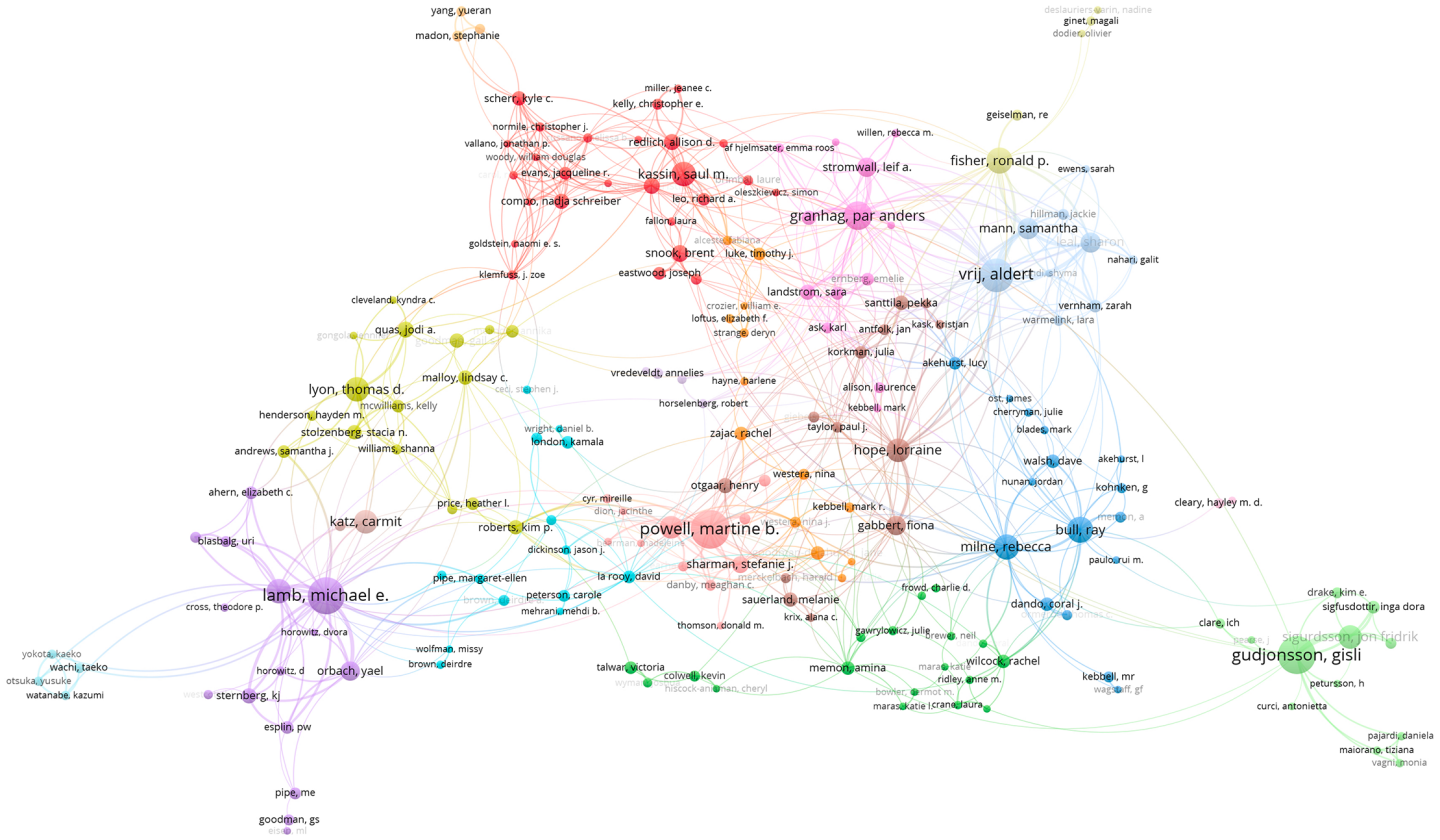
Co-authorship network (VOS Viewer).

**Figure 2:**
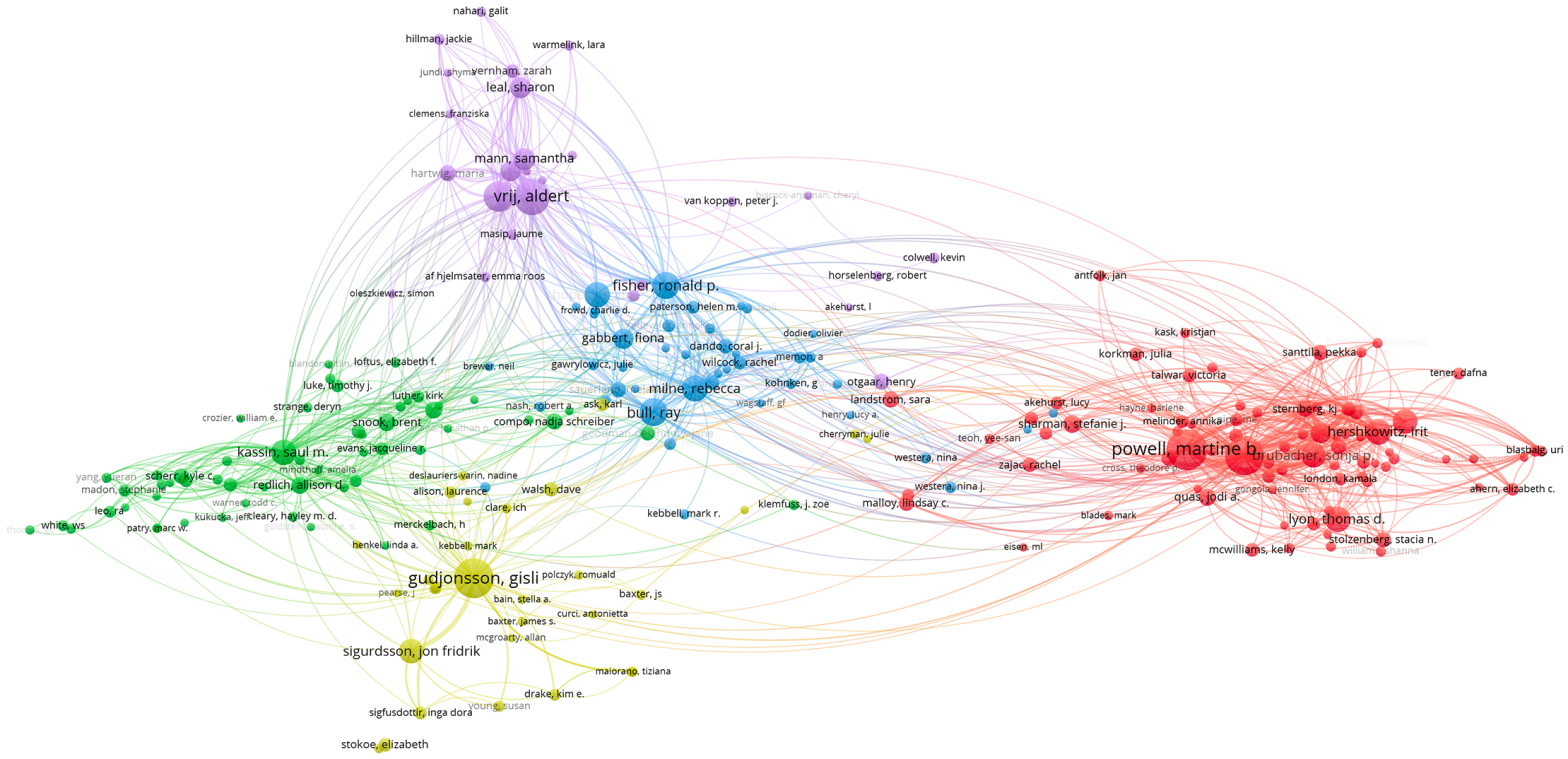
Citation (Authors) network (VOS Viewer).

Subsequently, to gain insights on collaboration patterns in the research field of criminal interrogations and investigative interviews, we exported our database in VOS Viewer and established the co-authorship network (see Figure 1) and the citation network, that is, who cites who (see Figure 2) of the 3,259 articles of our database on criminal interrogations and investigative interviews. However, to facilitate the understanding of those networks, VOS Viewer only considered authors who had 5 articles or more, and for the co-authorship network, also ignored articles that had 25 authors or more.

### The institutions

As shown in Table 7, the University of Portsmouth has the highest number of articles on criminal interrogations and investigative interviews is (*n* = 110). This is unsurprising considering five of the Top 15 authors (Aldert Vrij, Lorraine Hope, Samantha Mann, Sharon Leal, Rebecca Milne) are affiliated with that institution. King’s College London (*n =* 86), home of Gisli H. Gudjonsson, Deakin University (*n =* 83), previous home of Martine B. Powell, and follow closely at the second and third position. In contrast with the research field of deception and lying, no institution clearly stands out in the research field of criminal interrogations and investigative interviews. In the research field of deception and lying, for example, the University of Portsmouth, the institution in first place (*n =* 173), had around three times the number of articles of the University of Arizona, the institution in the second place (*n =* 67) (Denault et al., 2022). This is not the case in the research field of criminal interrogations and investigative interviews. It should be noted that academics in the research field of criminal interrogations and investigative interviews come from more than 1,000 institutions, emphasizing the magnitude of the scientific community.

**Table 7:** Top 15 institutions (of corresponding authors).

	Institutions	Number of articles	Percentage of all articles
1	University of Portsmouth	110	3.38
2	King’s College London	86	2.64
3	Deakin University	83	2.55
4	University of Gothenburg	67	2.06
5	University of Cambridge	51	1.56
6	Maastricht University	49	1.50
7	CUNY John Jay College of Criminal Justice	45	1.38
8a	Florida International University	40	1.23
8b	Griffith University	40	1.23
9	Tel Aviv University	34	1.04
10a	Memorial University of Newfoundland	28	0.86
10b	University of California Irvine	28	0.86
11	NICHD	25	0.77
12	University of Southern California	24	0.74
13	University of Michigan	22	0.68
14a	University of Otago	21	0.64
14b	University of Liverpool	21	0.64
15	University of Leicester	20	0.61

### The countries

Researchers interested in criminal interrogations and investigative interviews come from more than 50 countries, once again showing the magnitude of the scientific community. The first place goes to the United States (*n* = 1,136), with around two times the number of articles from the United Kingdom (*n* = 712) in second place, and around five times the number of articles from Australia (*n =* 229) in third place. However, since our database only features articles in English, the actual contribution of other countries, especially where English is not the primary language (e.g., Germany, France, Japan, Spain), is certainly underestimated (see Table 8).

**Table 8:** Top 15 countries (of corresponding authors).

	Countries	Number of articles	Percentage of all articles
1	United States	1,136	34.86
2	United Kingdom	712	21.85
3	Australia	229	7.03
4	Canada	190	5.83
5	Sweden	132	4.05
6	Netherlands	122	3.74
7	Israel	67	2.06
8	Germany	52	1.60
9a	Italy	47	1.44
9b	Norway	47	1.44
10	New Zealand	40	1.23
11	France	32	0.98
12	Finland	30	0.92
13	Belgium	29	0.89
14	Japan	23	0.71
15	Spain	21	0.64

### The keywords

**Figure 3:**
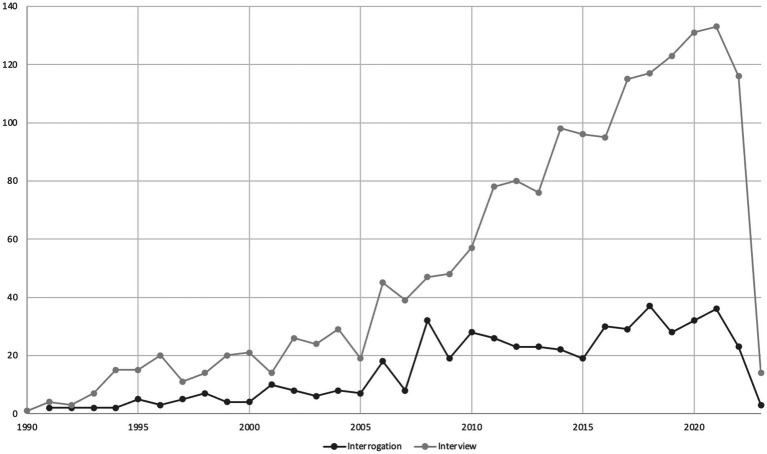
The words “Interrogation” and “Interview” in keywords per year.

As shown in Table 9, the Top 15 most used keywords show the variety of themes the scientific community is interested in. The results suggests that “memory” is the theme related to criminal interrogations and investigative interviews receiving the most attention from the scientific community. However, apart from memory, the number of articles from one keyword to another are similar, and no other theme clearly stands out in the research field of criminal interrogations and investigative interviews. It should be noted that the 10th keyword in the Top 15 most used keywords suggests, once again, that the scientific community is concerned about children. This was previously evident also in the Top 15 journals (see Table 3), and is confirmed by the Top 15 most cited articles (see Table 6). Finally, although the number of articles using the keyword “interrogation” appears in the fourth place, the number of articles using the keywords “interview” or “interviews” is higher, and the use of those words in keywords per year shows a higher increase for the latter (see Figure 3). This is unsurprising because law enforcement moved away from accusatorial methods which is often associated with the former. It should be noted that “nonverbal behavior” is in 95th position.

**Table 9:** Top 15 most used keywords (author’s keywords and keywords plus).

	Keywords	Number of articles	Percentage of all articles
1	Memory	550	16.88
2	Suggestibility	394	12.09
3	Sexual Abuse	309	9.48
4	Interrogation	302	9.27
5	Cognitive interview	285	8.75
6	Interviews	269	8.25
7a	Recall	260	7.98
7b	Accuracy	260	7.98
8	Eyewitness memory	259	7.95
9	False confessions	256	7.86
10	Children	240	7.36
11a	Interview	226	6.93
11b	Witnesses	226	6.93
12	Interrogative suggestibility	214	6.57
13	Confessions	207	6.35
14	Deception	201	6.17
15	Forensic Interviews	198	6.08

**Figure 4:**
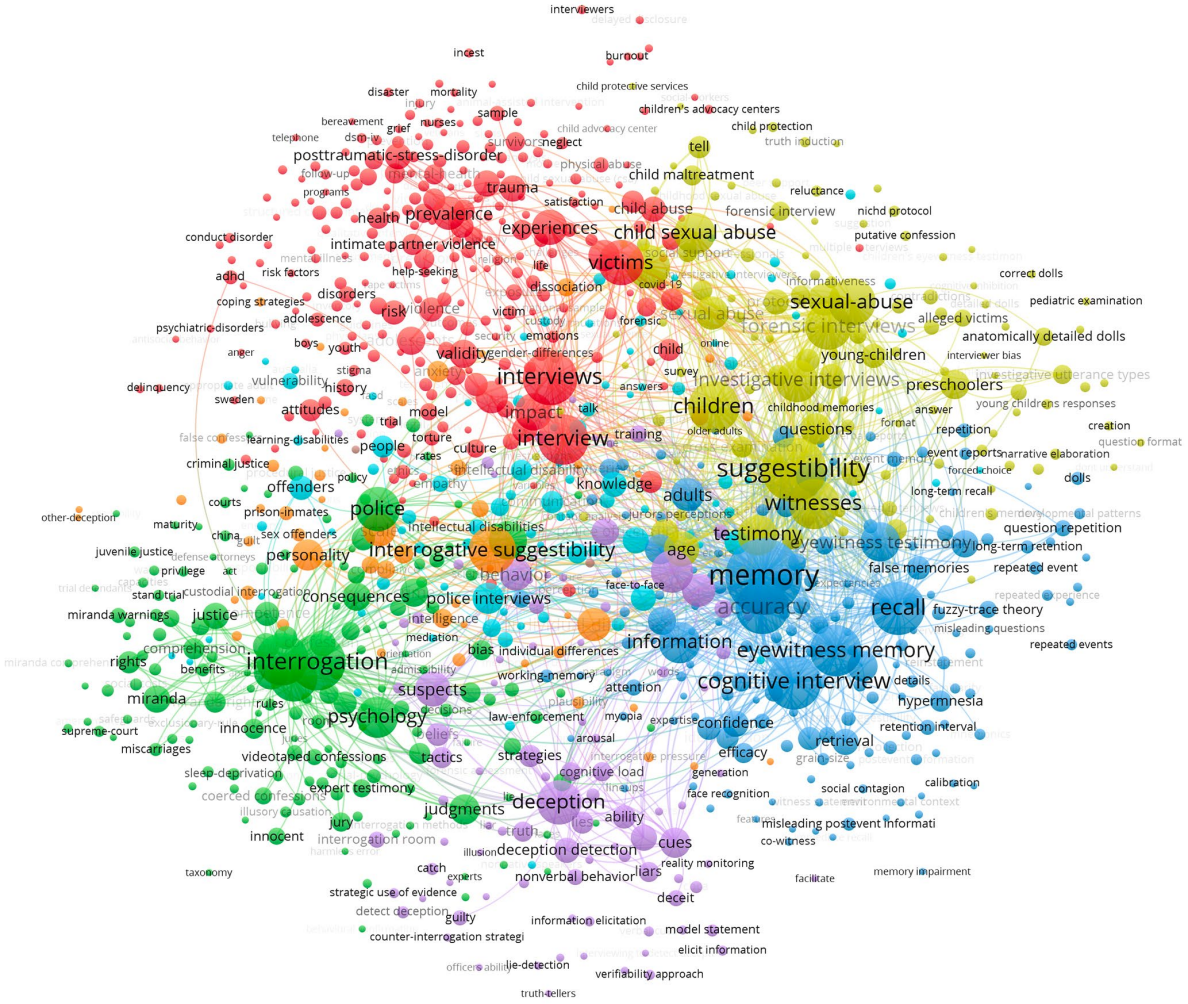
Keywords co-occurrence analysis (VOS Viewer).

Subsequently, to gain insights on thematic groups in the research field of criminal interrogations and investigative interviews, we exported our database in VOS Viewer and established the co-occurrence network (see Figure 4) of the 3,259 articles of our database on criminal interrogations and investigative interviews. However, to facilitate the understanding of this network, VOS Viewer only considered keywords who had 5 occurrences or more.

Furthermore, to have a better idea of the variety of themes the scientific community is increasingly interested in, the authors’ keywords and Keywords Plus were extracted for all articles published since 2013 (see Table 10), and from 2003 to 2012 (see Table 11). The results suggests that “memory” and “suggestibility” are still the two themes related to criminal interrogations and investigative interviews with the most attention from the scientific community. The results also suggest that the scientific community have become increasingly concerned about children, but less about confession, false confession, and eyewitness memory, among other things.

**Table 10:** Top 15 most used keywords since 2013.

	Keywords	Number of articles	Percentage of all articles
1	Memory	303	9.30
2	Suggestibility	185	5.68
3	Sexual Abuse	184	5.65
4	Interrogation	174	5.34
5	Cognitive Interview	167	5.12
6	Interviews	165	5.06
7	Children	160	4.91
8	Accuracy	150	4.60
9	Disclosure	145	4.45
10	False Confessions	139	4.27
11	Forensic Interviews	135	4.14
12a	Witnesses	132	4.05
12b	Eyewitness Memory	132	4.05
13a	Recall	128	3.93
13b	Deception	128	3.93
14a	Victims	127	3.90
14b	Interview	127	3.90
15	Investigative Interviewing	126	3.87

**Table 11:** Top 15 most used keywords from 2003 to 2012.

	Keywords	Number of articles	Percentage of all articles
1	Memory	157	4.82
2	Suggestibility	153	4.69
3	False Confessions	99	3.04
4	Interrogation	98	3.01
5a	Recall	86	2.64
5b	Eyewitness Memory	86	2.64
6	Interrogative Suggestibility	83	2.55
7	Accuracy	82	2.52
8a	Interviews	81	2.49
8b	Cognitive Interview	81	2.49
9	Sexual Abuse	77	2.36
10	Psychology	64	1.96
11	Deception	61	1.87
12a	Forensic Interviews	59	1.81
12b	Confessions	59	1.81
13a	Witnesses	57	1.75
13b	Interview	57	1.75
13c	Children	57	1.75
14	Testimony	53	1.63
15	Individual Differences	52	1.60

### The articles

The Top 15 most cited articles show a variety of topics related to criminal interrogations and investigative interviews (see Table 6). In the most cited article (*n* = 741), Harrison and Horne (2000) present a review of the impact of sleep deprivation on decision making, a topic relevant to police investigations, but also relevant to issues outside criminal interrogations and investigative interviews, which may have increased citation count. In the second most cited (*n* = 385), Saul M. Kassin and Gisli H. Gudjonsson, two authors from the Top 15 (see Table 5), and their colleagues present a review of the risk factors for police-induced confessions and offer recommendations to protect vulnerable individuals during criminal interrogations. Then, the third (London et al., 2005), fourth (Lamb et al., 2007), fifth (Fink et al., 1995), sixth (Bruck and Ceci, 1999), eighth (Orbach et al., 2000) and twelfth (Chard, 2005) articles address children related topics. The seventh article (Kassin and Kiechel, 1996) reports data showing that individuals may accept guilt for a crime they did not commit if presented with false incriminating evidence, and the eight article (Memon et al., 2010) present a meta-analytic review on the cognitive interview and a study space analysis of the past 25 years. At the 9th position, Exline et al. (2003) present a review about forgiveness, and Gudjonsson (1984) presents a scale on individual susceptibility to suggestion. At the 10th position, Meissner and Kassin (2002) report data showing that training and experience in deception detection fail to improve deception detection ability, and that experience in law enforcement may result in a bias where others are confidently, but erroneously judged more guilty. At the 11th position, Kassin (2005) presents a review on police practices that increase the risks of innocent individuals making false confessions, and at the 12th position, Chard evaluates a therapy for sexual abuse survivors. Finally, at the 13th position, Vrij, Mann, Fisher, Leal, Milne, and Bull, six authors from the Top 15 (see Table 5) report data showing that instructing suspects to report their stories in reverse order improve police observers’ deception detection ability (Vrij et al., 2008), at the 14th position, Vrij (2005) presents a review of research on the Criteria-Based Content Analysis, and at the 15th position, Geiselman et al. (1985) compare the effectiveness of interviewing procedures to improve the eyewitnesses memory.

### The research on questionable tactics and techniques

Despite the wealth of scientific knowledge on criminal interrogations and investigative interviews, as evidenced by our bibliometric study, a number of law enforcement organizations have turned to questionable tactics and techniques (e.g., Denault et al., 2020; Smith, 2020). But as some of them explicitly claim or implicitly suggest these techniques have widespread approval from the scientific community, the question arises: what is their weight in the research field of criminal interrogations and investigative interviews? The following five are examined: Kinesic Interview, Synergology, Scientific Content Analysis (SCAN), Behavior Analysis Interview (BAI), and Reid Technique. Walters (2002) defines Kinesic Interview:

Kinesic interview and interrogation is viewed as a multiphase behavioral analysis system used to conduct more effective and efficient interpersonal communications… speech and body language behaviors can give insight into the individual’s personality type, indicating the “psychological fingerprint” of that person. By combining the information received through diagnosis of verbal and nonverbal behavior with this psychological fingerprint, an interviewer can conduct an interview and interrogation that is specifically tailored for the subject (p. 2-3).

Even if it bears the hallmarks of a pseudoscience (Denault, 2020; Denault et al., 2020), kinesics interview is popular among various organizations, including the Association of Certified Fraud Examiners (ACFE) who promote it in their Fraud Investigators Manual, “the definitive body of knowledge for the anti-fraud profession, providing comprehensive guidance for anti-fraud professionals that no other work can match” (ACFE, n.d.). However, following a search in our database (titles, abstracts or keywords) not one article in our corpus focuses on Kinesic Interview.

According to its proponents, synergology is a “scientific discipline for reading gestures” (Synergology, The Official Website, n.d.). However, only one article in our corpus refers to synergology where it is described as a problematic method within security and justice contexts (Denault et al., 2020). And only one article (Armistead, 2011) in our corpus refers to Scientific Content Analysis (SCAN), and it addresses deficiencies of a study supporting SCAN as a technique to analyze textual documents.

Behavior Analysis Interview (BAI), however, is addressed in 7 articles. Three with Aldert Vrij as the first authors, three with Jaume Masip as the first author, and one with Vincent Denault as the first author. Masip et al. (2011) report data showing BAI recommendations are inaccurate and promote common-sense beliefs, Masip et al. (2012) replicate those findings with law enforcement officers, and Masip and Herrero (2013) report data questioning the value of BAI in identifying perpetrators. Vrij et al. (2006) report data opposing predictions of BAI, Vrij et al. (2007) report data showing BAI’s standardized list of 15 questions are useless to detect lies using verbal or nonverbal behavior, and Vrij and Fisher (2016) argues that BAI cannot be included in a standard investigative interview. Finally, Denault et al. (2020) is the same article addressing synergology. BAI is described as a problematic method within security and justice contexts.

Finally, the Reid Technique is addressed in 12 articles. In the first article and second article, King and Snook (2009) reports data showing how the Reid Technique is used in video-recorded police interrogations in Canada, and Kostelnik and Reppucci (2009) report data showing police officers trained in the Reid Technique are less sensitive to the development maturity of adolescents and use a number of psychologically coercive tactics with adolescents. In the third article, Gallini (2010) argues that “From a policy standpoint, continued reliance on the Reid technique does a disservice to our justice system and unnecessarily risks obtaining inherently unreliable confessions. From an evidentiary standpoint, the methodology underlying the Reid technique fails every aspect of the Supreme Court’s standards governing the admission of expert evidence” (p. 529). In the fourth, Gudjonsson and Pearse (2011) review police commonly used interviewing methods and their potential for false confessions, and in the fifth, Perri (2011) mentions the Reid Technique appears to have been used in the flawed interview of a convicted killer. The sixth article (Cleary and Warner, 2016) reports data showing experienced police officers are often trained in (legally permissible) psychologically coercive tactics, similarly with adult and juvenile interviewees, and the seventh article (Mason, 2016) present a case study addressing strategies embedded in the Reid Technique used by police officers to pressure suspects into cooperation. The eight article (Luke et al., 2017) reports data on how the bait question, a type of question promoted by the Reid Technique, can distort the memory of suspects. In the ninth article, Spierer (2017) argues that “The coercion and deception inherent in the Reid Technique, coupled with the recognized vulnerabilities and susceptibilities of children as a group, has led to an unacceptably high rate of false confessions among juvenile suspects. And, when a juvenile falsely confesses as the result of coercive interrogation tactics, society ultimately suffers a net loss” (p. 1719). In the tenth article, Keatley et al. (2018) report, among other things, on how the Reid Technique can result in suspects providing (false) confessions. Finally, in the 11th article, French (2019) address “how the courts’ outdated understanding of coercion has impacted the evaluation of confession evidence and fueled the continued existence of the Reid accusatory model of interrogation” (p. 1031), and in the 12th article, Snook et al. (2020) provides a critical analysis of the Royal Canadian Mounted Police’ Phase Interview Model, and explains how it features strategies of the Reid Technique.

## Discussion

The research field of criminal interrogations and investigative interviews fundamentally changed police practices (Kassin et al., 2010). However, little was known about the structure of this research field. This paper presented the results of a bibliometric study providing a comprehensive overview of the social science research conducted on criminal interrogations and investigative interviews since the 1900s. The results revealed the richness of the research field of criminal interrogations and investigative interviews. Firstly, in the 1990s, this research field took off strongly. Secondly, issues of criminal interrogations and investigative interviews are subject to thousands of articles, written by thousands of researchers, published in hundreds of journals. Thirdly, the research areas, universities and countries (more than 50) interested this research field show the magnitude of the scientific community. Finally, keywords show the variety of themes the scientific community is interested in, “memory” and “suggestibility” receiving the most attention. The results of our bibliometric study also suggest the scientific community have become increasingly concerned about children, but less about confession, false confession, and eyewitness memory.

The richness of the research field of criminal interrogations and investigative interviews highlights what practitioners miss out when turning to unfounded and discredited tactics and techniques (e.g., Denault et al., 2020; Smith, 2020). To help practitioners understand the breadth of scientific knowledge on criminal interrogations and investigative interviews, if it takes four months to create an article (a very conservative estimate), from data collection to manuscript writing, it would take about 271 years to publish the 3,259 articles with a team of four researchers. Therefore, why have a number of law enforcement organizations turned to unfounded and discredited tactics and techniques rather than evidence-based practices?

Law enforcement has an history of tactics and techniques lacking scientific support, inside, but also outside the interrogation room (e.g., Lilienfeld and Landfield, 2008). For example, in the United States, microscopic hair analysis was used to identify suspects in countless investigations. But in 2015, “the FBI has concluded that the examiners’ testimony in at least 90 percent of trial transcripts the Bureau analyzed as part of its Microscopic Hair Comparison Analysis Review contained erroneous statements” (FBI, 2015). Additional examples of tactics and techniques lacking scientific support include the identification of criminals from bitemark patterns (National Institute of Standards and Technology, 2023), and the use of 911 calls to arrest, prosecute and convict individuals (Murphy, 2022). This popularity, although worrying, is not surprising. As argued by Denault et al. (2020), so-called experts “offer immediate and easy solutions to complex challenges” (p. 7). Moreover, when organizations are faced with problems to solve, the lack of scientific knowledge, the ignorance of the importance of science, and the underestimation of the dangers of pseudoscience makes them vulnerable to unfounded and discredited tactics and techniques. But the scientific community also bears some responsibility for why a number of law enforcement organizations have turned to tactics and techniques lacking scientific support.

Researchers and practitioners interested in criminal interrogations and investigative interviews must be proactive in communicating research findings, explaining why laboratory studies, even if they cannot always fully capture the complexity of actual interviews, are relevant for law enforcement, but also in addressing questionable tactics and techniques, and in helping law enforcement in recognizing and resisting to misinformation (Ecker et al., 2022). Although by its very nature scientific knowledge is constantly evolving, and there is always a need for further research, it is fundamental to take research findings, as they stand, with their strengths and limitations, and improve tactics and techniques for solving crimes and identifying perpetrators, even if it means improving them again when research findings change. Because otherwise, so-called experts will “offer immediate and easy solutions to complex challenges” (Denault et al., 2020, p. 7), and by the time research findings are disseminated, the questionable tactics and techniques will be overly rooted in organizations. Because of reputational damages and lawsuits they may face if they change their tactics and techniques, thereby admitting, either implicitly or explicitly, that previous tactics and techniques were inadequate, organizations might stand their ground. This will impede the dialog between researchers and practitioners. And eventually, even if organizations change their tactics and techniques, and practitioners learn that their beliefs are inaccurate, the continuous misinformation effect (Lewandowsky et al., 2012) will limit the impact of the change. In other words, even after practitioners learn their beliefs are inaccurate, their beliefs still influence them.

In addition, the richness of the research field of criminal interrogations and investigative interviews highlights the negligible contribution of questionable tactics and techniques to the literature on criminal interrogations and investigative interviews. That is, in addition to being subject to scrutiny in peer-reviewed publications, the questionable tactics and techniques lack widespread approval from the scientific community, contrary to what their proponents explicitly claim or implicitly suggest. For example, “nonverbal behavior” being far down in the list of the most used keywords, tactics and techniques focusing on “nonverbal behavior,” and sold as being the best for solving crimes and identifying perpetrators, should arouse suspicion. However, nonverbal behavior is vitally important to investigative interviews, as it is with any other face-to-face interaction. But not just as it is professed on social medias and television shows such as Lie To Me. As Hall et al. (2019) underline,

The breadth of topics that relate to NVC is quite wide, in accordance with its many functions, which include displaying affect (such as anxiety or happiness), revealing attitudes (such as interest, prejudice, or intimacy), regulating interaction (such as taking turns or directing attention), managing impressions (such as by presenting oneself as competent or brave), revealing physical and mental conditions (such as pain or mental disorders), and exerting interpersonal control (as in displaying dominance) (p. 273).

Therefore, researchers and practitioners interested in criminal interrogations and investigative interviewing should beware of falling into the trap of overlooking the vital importance of the nonverbal behavior because questionable tactics and techniques are heavily promoted by so-called experts. Rapport building, for example, in central in eliciting the truth (Meissner et al., 2012), and nonverbal behavior is central in rapport building (Tickle-Degnen and Rosenthal, 1990). This makes nonverbal behavior central in solving crimes and identifying perpetrators.

Finally, despite the wealth of scientific knowledge on criminal interrogations and investigative interviews, as evidenced by our bibliometric study, the research community should keep in mind investigations are at the very beginning of the judicial process. Subsequently, if there is a trial,^1^ the judge in a bench trial, or the jurors in a jury trial, will evaluate the evidence. And this should be considered by the research community. As Vrij and Granhag (2012) highlighted,

… researchers must provide criminal investigators with techniques that will help them to produce evidence that will stand up in court. It is not just about assessing whether a suspect is lying or telling the truth, it is also about maximising the value of the evidence so that prosecutors can present it ‘beyond reasonable doubt’, the standard of proof typically required in criminal courts (p. 115).

In other words, beyond solving crimes and identifying perpetrators, if tactics and techniques do not stand up to the tests of the courts, and do not provide evidence of high value, research funding (paid for by public taxes) that supported their development will have a limited impact on the civil society. The same holds if, ultimately, courts assess the credibility of witnesses based on stereotypes and prejudices (Denault et al., 2023), and do not give appropriate weight to the evidence collected with state-of-the-art tactics and techniques.

## Conclusion

Our bibliometric study provided a rigorous, and novel picture of this research field. However, our bibliometric study is not without limitation. For example, as mentioned above, it focuses on a particular strand of research, that is, social sciences research on criminal interrogations and investigative interviews. In other words, the use of other databases, and of other keywords might yield different results, and as Denault et al. (2022) pointed out, the keywords to create the corpus of articles for our bibliometric study could be subject to debate. Furthermore, many articles that do not deal with investigative interviews, but whose focus is relevant to investigative interview, may have an important role in developing better police practices, are not featured in our database. For example, peer-reviewed publications on lie detection, nonverbal behavior, memory, and cognitive biases, even if not addressing investigative interviews, can be of great importance for investigative interviews. Our bibliometric study, like others have done before (e.g., Nadeau et al., 2018; Plusquellec and Denault, 2018; Dodier, 2019; Denault et al., 2022), shows that, in the end, even if research fields are intuitively independent, even if researchers work in silos, research findings transcends disciplinary boundaries, and embracing interdisciplinary research can only foster the development of better police practices to solve crimes and identify perpetrators.

## Author contributions

All authors listed have made a substantial, direct, and intellectual contribution to the work and approved it for publication.

## Acknowledgments

The authors would like to thank Vincent Larivière for his constructive comments on an earlier version of this article.

## Conflict of interest

The authors declare that the research was conducted in the absence of any commercial or financial relationships that could be construed as a potential conflict of interest.

## Publisher’s note

All claims expressed in this article are solely those of the authors and do not necessarily represent those of their affiliated organizations, or those of the publisher, the editors and the reviewers. Any product that may be evaluated in this article, or claim that may be made by its manufacturer, is not guaranteed or endorsed by the publisher.

## Footnotes

^1^It should be mentioned that even if the strength of the evidence can influence defendants in pleading guilty, and therefore, avoid a trial, a number of defendants plead guilty for crimes they did not commit (Gross, 2008; Dervan and Edkins, 2013; Blume and Helm, 2014).
